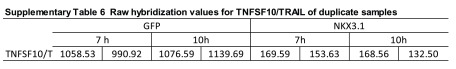# Systems analysis of the prostate tumor suppressor NKX3.1 supports roles in DNA repair and luminal cell differentiation

**DOI:** 10.12688/f1000research.3818.2

**Published:** 2014-12-18

**Authors:** Chih-Cheng Yang, Alicia Chung, Chia-Yu Ku, Laurence M. Brill, Roy Williams, Dieter A. Wolf

**Affiliations:** 1 Tumor Initiation and Maintenance Program, Sanford-Burnham Medical Research Institute, La Jolla, CA 92037, USA; 2 NCI-designated Cancer Center Proteomics Facility, Sanford-Burnham Medical Research Institute, La Jolla, CA 92037, USA; 3 Informatics and Data Management Core, Sanford-Burnham Medical Research Institute, La Jolla, CA 92037, USA; 4 San Diego Center for Systems Biology, La Jolla, CA 92093-0375, USA; 5 Genentech Inc., South San Francisco, CA 94080, USA

## Abstract

NKX3.1 is a homeobox transcription factor whose function as a prostate tumor suppressor remains insufficiently understood because neither the transcriptional program governed by NKX3.1, nor its interacting proteins have been fully revealed. Using affinity purification and mass spectrometry, we have established an extensive NKX3.1 interactome which contains the DNA repair proteins Ku70, Ku80, and PARP, thus providing a molecular underpinning to previous reports implicating NKX3.1 in DNA repair. Transcriptomic profiling of NKX3.1-negative prostate epithelial cells acutely expressing NKX3.1 revealed a rapid and complex response that is a near mirror image of the gene expression signature of human prostatic intraepithelial neoplasia (PIN). Pathway and network analyses suggested that NKX3.1 actuates a cellular reprogramming toward luminal cell differentiation characterized by suppression of pro-oncogenic c-MYC and interferon-STAT signaling and activation of tumor suppressor pathways. Consistently, ectopic expression of NKX3.1 conferred a growth arrest depending on TNFα and JNK signaling. We propose that the tumor suppressor function of NKX3.1 entails a transcriptional program that maintains the differentiation state of secretory luminal cells and that disruption of NKX3.1 contributes to prostate tumorigenesis by permitting luminal cell de-differentiation potentially augmented by defects in DNA repair.

## Introduction

NKX3.1 encodes a homeodomain transcription factor whose expression is largely restricted to the prostate and controlled by androgen. The gene is located on chromosome 8p21 in a region frequently deleted in early prostate cancers (reviewed in ^1, 2^). Studies in Nkx3.1 knockout mice have provided compelling evidence that Nkx3.1 is a prostate tumor suppressor ^3– 5^. These mice develop prostatic intraepithelial neoplasia (PIN), a precancerous lesion characterized by hyperproliferation of dysplastic cells, indicating that Nkx3.1 is haploinsufficient for PIN suppression ^6^. Additional studies showed that serial passage of PIN-like lesions from Nkx3.1 mutant mice can undergo progressively severe histopathological alterations ^5^. Finally, loss of Nkx3.1 can cooperate with loss of Pten and p27 in prostate cancer development in mice ^7, 8^, while Nkx3.1 overexpression inhibits cell proliferation in Pten null epithelial grafts ^9^. These data indicate that the diminished expression of NKX3.1 that is frequently observed in human prostate cancers ^10^ is involved in the initial stage of prostate carcinogenesis. While the tumor suppressor function of NKX3.1 remains poorly defined at the molecular level, the knockout phenotypes suggested that Nkx3.1 controls genes involved in prostate development, differentiation, and maintenance of tissue integrity.

Like other NKX class homeoproteins, NKX3.1 can function as a transcriptional repressor by binding a non-canonical homeodomain DNA motif such as naturally occurring in the mouse androgen receptor promoter ^9^ or artificially presented in synthetic reporter genes ^11^. Transcriptional repression may involve NKX3.1-mediated recruitment of co-repressors ^12^ and the histone deacetylase, HDAC1 ^9^. A second mode of trans-repression found for the prostate-specific antigen (PSA) gene occurs independently of NKX3.1 promoter binding sites, but through repressive interaction with transcriptional activators such as SP1 ^13^ and prostate-derived ETS factor (PDEF ^14^). NKX3.1 was also shown to activate gene transcription, either through direct promoter binding as in the case of PCAN1 and HK2 ^15, 16^ or through interaction with other transcriptional activators such as serum response factor (SRF) or FoxA1 and the androgen receptor (AR) ^17, 18^.

Transcriptomic profiling combined with global mapping of > 9,500 genomic binding sites by ChIP-sequencing revealed a set of 282 putative direct target genes that were differentially expressed in young NKX3.1 ^-/-^ prostates not displaying PIN ^16, 19^. A subset of NKX3.1 target genes was also regulated by Myc with both transcription factors showing mutual antagonism ^16^. Since overexpression of Myc cooperates with loss of Nkx3.1 in mouse prostate tumorigenesis, maintaining proper control of the common Nkx3.1/Myc target genes may be involved in Nkx3.1’s tumor suppressor function ^16^. A similar study in aged mice already displaying PIN revealed a gene expression signature indicative of impaired response to oxidative stress ^20^. Interestingly, these changes correlated with a 5-fold increase in oxidative DNA damage in Nkx3.1 ^-/-^ prostates. Whether oxidative DNA damage is a direct consequence of loss of NKX3.1 or a secondary consequence of PIN development is unknown.

Another key to understanding the tumor suppressor function of NKX3.1 potentially lies with its protein interaction partners. Several have been described that modulate NKX3.1’s transcriptional effects (e.g. SRF ^17, 21^, PDEF ^14^, HDAC1 ^9^, SP1 ^13^, MYC ^16^, and AR ^18^). In addition, NKX3.1 was shown to bind to and augment the activity of topoisomerase I, suggesting that it functions in DNA repair ^22, 23^. NKX3.1 localizes to sites of DNA damage, promotes ATM and ATR activity, and enhances the survival of cells exposed to DNA damage ^24^. Loss of NKX3.1 function in premalignant prostate cells may therefore accelerate the acquisition of DNA damage, potentially aggravated by unabated accumulation of reactive oxygen species thus promoting cellular transformation ^24^. Nevertheless, it is currently unclear whether the function of NKX3.1 in DNA repair is indirectly mediated through transcriptional effects or directly through physical interactions with the DNA repair machinery.

In this report, we present an analysis of the NKX3.1 protein interactome that revealed intimate physical links of NKX3.1 with the DNA repair machinery, namely components of the DNA-dependent protein kinase (DNA-PK) holocomplex (XRCC5/Ku80, XRCC6/Ku70) and poly(ADP) ribose polymerase (PARP1). In addition, transcriptomic profiling of immortalized prostate epithelial cells upon acute activation of NKX3.1 revealed a rapid and complex transcriptional response that is a near mirror image of the gene expression signature of human PIN devoid of NKX3.1. Taken together, these data shed new light onto the elusive tumor suppressor activity of NKX3.1, directly implicating this homeoprotein in DNA repair and in driving a gene expression signature indicative of an essential function in maintaining the differentiation state of luminal prostate epithelial cells.

## Materials and methods

### Tissue culture, plasmids, viruses, antibodies

The human prostate cancer cell line LNCaP was obtained from ATCC and maintained in RPMI 1640 (Hyclone, Cat.# SH30027.01) supplemented with 10% fetal bovine serum (Sigma, Cat.# F6178-500ML), 50 units/ml penicillin, and 50 units/ml streptomycin (Thermo Scientific HyClone, Cat.# SV30010). The NKX3.1 cDNA was amplified from LNCaP mRNA, sequence confirmed, and cloned into pFLAG thereby attaching three consecutive FLAG epitope tags to the N-terminus. For DNA transfection, LNCaP cells were grown to 50–70% confluence on a 150 mm dish and transfected with 30 µg of plasmid DNA using DOTAP reagent according to the recommendations of the manufacturer (Roche, Indianapolis, IN). Immortalized human prostate epithelial cells (LH cells, kindly provided by Dr. W. Hahn; ^25^) were maintained in Prostate Epithelial Cell Basal Media (Lonza, Cat.# CC-3165) including growth factors, cytokines, and supplements (PREGM Singlequots, Lonza, Cat. # CC-4177).

For production of adenoviruses, the ADEASY system was used as previously described ^26^. The NKX3.1 cDNA was cloned into the pADTRACK1 shuttle vector. The resulting plasmid was transformed into BJ-ADEASY cells by electroporation. Adenoviral DNA generated by recombination in BJ-ADEASY cells was isolated and transfected into 293 cells (ATCC) using standard calcium phosphate procedures. Virus was harvested from cells and amplified by infection of 293 cells. Amplified virus was tittered and used at a multiplicity of infection of ~100.

The following antibodies were used: Flag mouse monoclonal (Sigma-Aldrich Cat# F1804, RRID:AB_262044), NKX3.1 mouse monoclonal for immunoblotting (Invitrogen Cat# 35-9700, RRID:AB_138690), Anti-human NKX3.1 goat polyclonal (Santa Cruz Biotechnology, Inc. Cat# sc-15022, RRID:AB_650285) for immunoprecipitation, GFP mouse monoclonal (Clontech Cat# 632380, RRID:AB_10013427), actin mouse monoclonal (MP Biomedicals, Irvine, CA, Cat.# ICN691001), BANF rabbit polyclonal (EMD Millipore Cat# 09-893, RRID:AB_1977041), Ku70 mouse monoclonal (GeneTex Cat# GTX23114, RRID:AB_367103), Ku80 mouse monoclonal (GeneTex Cat# GTX72225, RRID:AB_383445 ), MYC rabbit polyclonal (Epitomics Cat# 1472-1, RRID:AB_562270), p21 rabbit monoclonal (Cell Signaling Technology Cat# 2947S, RRID:AB_823586), HSPA8 rabbit polyclonal (Sigma-Aldrich Cat# SAB2101098, RRID:AB_10604580), PARP mouse monoclonal (BD Biosciences Cat# 556494, RRID:AB_396433), HOXB13 rabbit polyclonal (Invitrogen Cat# 422500, RRID:AB_1500227).

### FLAG-NKX3.1 affinity purification

Cells of one 150 mm dish transfected with pFLAF-NKX3.1 or empty vector were lysed in each 1 ml IP lysis buffer (50 mM Tris-HCl pH 7.4, 150 mM NaCl, 1% Triton X 100) on ice. Per affinity purification, 4 µg FLAG M2 antibody (Sigma-Aldrich Cat# F1804, RRID:AB_262044) was coupled to 50 μl magnetic beads in 0.2 M triethanolamine, pH 8.2 and 20 mM dimethyl pimelimidate with rotational mixing at room temperature for 30 min. The reaction was stopped by resuspending beads in 1 ml 50 mM Tris, pH 7.5 for 15 min. After five washes in IP lysis buffer, the beads were added to the cell lysate. Upon incubation for 4 h at 4°C, the lysate was removed and stored as “depleted lysates” at -20°C, whereas the beads were washed 5 times with 1 ml IP lysis buffer. After the final wash, beads were resuspended in 50 µl elution buffer (5 µg of triple FLAG peptide in PBS) and incubated at 4°C for 30 minutes with vortexing. The sample was analyzed by immunoblotting (10%), silver staining (2%), and LC-MS/MS (88%).

### Liquid chromatography and tandem mass spectrometry (LC-MS/MS)

LC-MS/MS analysis of affinity purified FLAG-NKX3.1 complexes was performed as previously described in detail ^27, 28^. In brief, eluates were digested in solution with trypsin, and peptides were separated by reversed phase chromatography. Peptides were analyzed on an LTQ Orbitrap XL mass spectrometer (Thermo Fisher Scientific; San Jose, CA). The MS/MS method was top 4-data dependent. Dynamic exclusion was enabled. Data were searched against an international protein index (IPI) human protein database using Sorcerer-SEQUEST (SageN Research; Milpitas, CA).

### Semi-quantitative analyses using spectral counting

Spectral counts are the number of times an ionized peptide is selected by the mass spectrometer for MS/MS, in the data-dependent mode and provide widely accepted, semi-quantitative estimates of relative protein abundance ^29^. QTools, which are in-house developed visual basic macros (available from: www.dieter-wolf-lab.org/protocols) for automated spectral count analysis, were used to compute spectral counts of the proteins, using the PeptideProphet output from the trans-proteomic pipeline (TPP; Institute for Systems Biology, Seattle, WA; ^30^).

### Post-identification protein filtering

Purifications of FLAG-NKX3.1 were performed in quadruplicate (i.e. 4 biological replicates), each time starting with a fresh batch of cells. Altogether eight samples from affinity purifications (quadruplicates of mock and FLAG-NKX3.1) were analyzed repeatedly (3 times per sample, i.e. 3 technical replicates of each sample) by LC-MS/MS for a total of 24 LC-MS/MS runs.

Altogether we identified 315 human proteins ( Data set 1A). To compile a high confidence NKX3.1 protein interactome, we first performed a background subtraction, i.e. the spectrum count obtained for each protein in the mock purifications was subtracted from the spectrum count obtained for that same protein in the corresponding FLAG-NKX3.1 purification ( Data set 1B). The subtracted spectrum counts were then summed over all 4 independent purifications. If negative values were obtained after summing (i.e. if a protein was consistently more abundant in the mock purification than in the FLAG-NKX3.1 purification), the protein was disregarded. This resulted in a list of 250 proteins with an average spectrum count of 9.94 ( Data set 1B). From this lists of background-subtracted data, we removed all proteins with spectrum counts below the average (≤ 10) to exclude low-abundance proteins potentially non-specifically associated with NKX3.1. This resulted in a list of 71 background subtracted and abundance-filtered proteins. In the next step, we collapsed redundant protein database entries (often resulting from multiple protein isoforms that were not distinguished by the peptides identified by LC-MS/MS) into single entries by adding their spectrum counts both in the mock and NKX3.1 purifications. This resulted in a non-redundant list of 58 proteins, which we refer to as the high confidence interactome ( Data set 1B).

**Figure 1.**
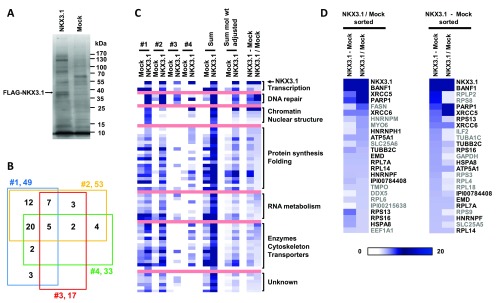
The NKX3.1 protein interactome. ( **A**) Representative purification of FLAG-NKX3.1 from transfected LNCaP cells. Cell lysates were absorbed to anti-FLAG M2 resin, and specifically retained proteins were eluted with FLAG peptide and separated by SDS-PAGE. A band migrating with the expected molecular weight of FLAG-NKX3.1 and absent from the mock purification (empty vector) is highlighted. ( **B**) Four-way Venn diagram to indicate the degree of overlap in the protein content detected in four independent purifications of FLAG-NKX3.1. ( **C**) Map of spectrum count intensities in the four independent FLAG-NKX3.1 and mock purifications. The map also contains the sum of spectrum counts across all purifications as well as summed data after adjustment for protein molecular weights. The right most two columns present two distinct ways of background correction, either by subtracting mock values from NKX3.1 values (NKX3.1 – Mock) or by calculating the factor of enrichment in the NKX3.1 sample over mock (NKX3.1/Mock). See the Materials and methods section for details on data analysis and processing. ( **D**) Spectrum count intensity maps of the 25 most abundant components of the NKX3.1 interactome. Data were sorted either by factor of enrichment (left panel, NKX3.1/Mock sorted) or by background subtracted values (right panel, NKX3.1 – Mock sorted). Black type font indicates the proteins occurring on both lists independent of the method of abundance-based sorting.

Since spectrum counts depend on protein size (larger proteins giving rise to more tryptic peptides), we normalized spectrum counts to protein molecular weights, which we have previously found to be an appropriate method of normalization ^31^. The summed, normalized spectrum count numbers of all non redundant proteins were used to assemble the final background subtracted list of 58 NKX3.1 interacting proteins (referred to as Sum NKX3.1 – Mock). The summed normalized spectrum count numbers were also used to determine the fold enrichment of a protein in the NKX3.1 sample over mock (Sum NKX3.1/Mock). Both lists were sorted according to abundance and compared in Figure 1D to illustrate that both methods of background filtering (subtraction or division) yield an overlapping list of high confidence NKX3.1 interactors. The spectrum count intensity map in Figure 1C reiterates most of the steps described above thus presenting a comprehensive view of the analysis.

### Reactome analysis

**Figure 2.**
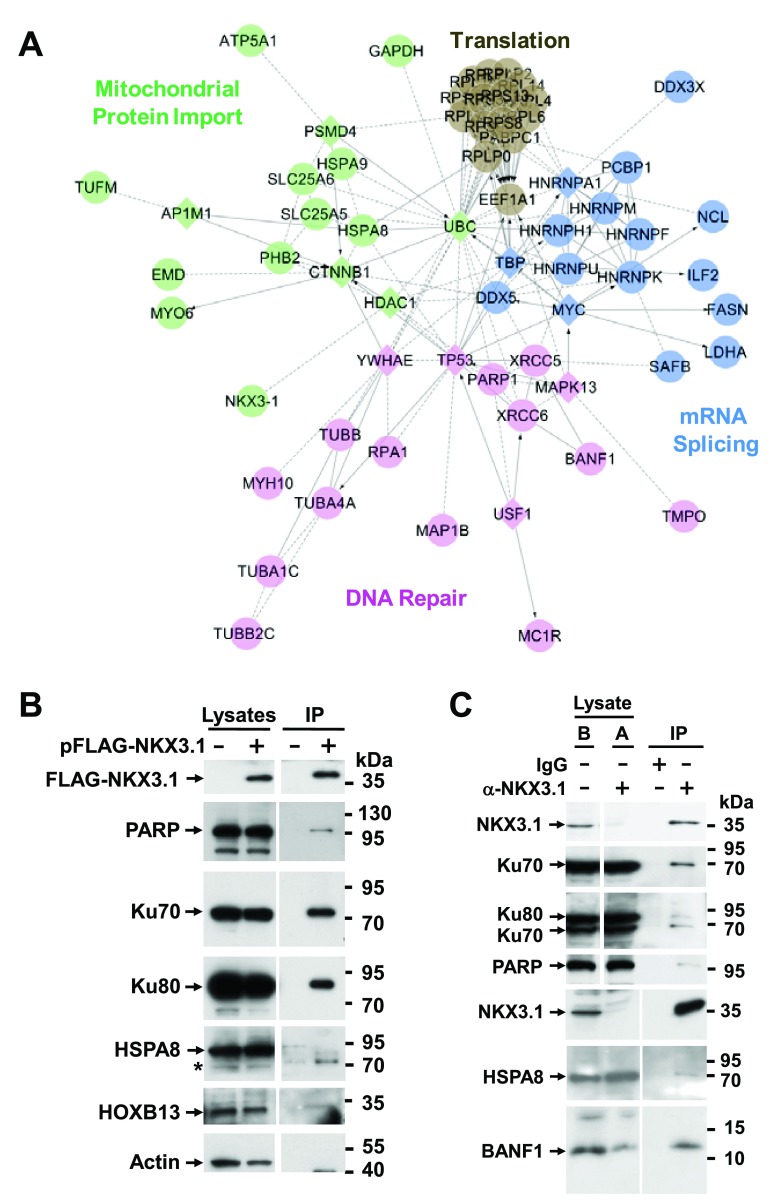
NKX3.1 interacts with DNA repair proteins. ( **A**) The list of NKX3.1 interacting proteins was loaded into Cytoscape and used to build Reactome Functional Interaction networks. The networks were clustered into modules (indicated by colors), and pathways enriched in the modules (FDR ≤ 0.01) were identified. Diamonds represent network components that were not identified as NKX3.1 interacting proteins. ( **B**) LNCaP cells were transfected with FLAG-NKX3.1 (+) or empty vector (-) followed by absorption of cell lysate to FLAG M2 resin to purify FLAG-NKX3.1. Co-purifying DNA repair proteins were detected by immunoblotting. The bottom four panels are from the same affinity purification resolved on a separate gel. The asterisk denotes an unspecific cross-reactivity of the HSPA8 antibody. Cropped blot images are shown; see Figure S7 for full images. ( **C**) A nuclear protein fraction was prepared from LNCaP cells and employed for immunoprecipitation with NKX3.1 antibodies or an IgG control as indicated. The same samples before (“B”) and after (“A”) immunoprecipitation are shown to document the specific depletion of endogenous NKX3.1. The bottom three panels are from the same immunoprecipitate resolved on a separate gel. Cropped blot images are shown; see Supplement Figure S7 for full images.

The NKX3.1 interactome was analyzed with the Cytoscape Reactome FI plugin ^32^. The list of NKX3.1 interacting proteins was loaded into Cytoscape and used to build Reactome networks allowing linker genes. The networks were clustered into modules, and pathways enriched in the modules (FDR ≤ 0.01) were identified ( Figure 2A).

### Transcriptome analysis

Duplicate RNA samples collected from NKX3.1 adenovirus transduced LH cells or from LH cells transduced with the GFP control virus were used for microarray analysis on the Illumina platform. The Human 6-V2 Expression BeadChips (Illumina) were used, which contain ~46,000 transcript probes. Primary data was collected using the manufacturer’s BeadArray Reader using the supplied scanner software. Data analysis was done in three stages. First, expression intensities were calculated for each transcript probed on the array for all hybridizations using Illumina’s Beadstudio#2 software. Secondly, intensity values were quality controlled and normalized. Quality control was carried out by using the Illumina Beadstudio detection p-value set to < 0.05 as a cutoff. This removed probes whose signals were too low to be reliably detected on the array. After this step, the initial ~46,000 probes were reduced to 22,319 ( Data set 2A). Measurements were then normalized using the *normalize.quantiles* routine from the Affymetrix package ^33^ in Bioconductor (version 2.5, R version 2.10.1). This procedure accounted for any variation in hybridization intensity between the individual arrays. An assessment of several different normalization techniques using the Bioconductor *maCorrPlot* routine suggested that *normalize.quantiles* was the most appropriate for the data. Finally, these normalized data were imported into GeneSpring and analyzed for differentially expressed genes. The raw datasets were submitted to the GEO database (accession number GSE47030).

To identify genes differentially expressed between LH cells infected with Ad-GFP and Ad-GFP-NKX3.1 the biological replicates for each time point (7 h and 10 h) were averaged. Datasets were interrogated for genes with statistically significant differences between the two groups (i.e. +/- NKX3.1) based on the results of the Welch t-test (parametric test, variances not assumed equal; p-value cutoff 0.05). To find the genes with the most robust changes in expression, the data was plotted as a “Volcano Plot” ( Supplementary Figure S2B), which allows statistical significance to be measured along with the extent of fold change in expression. Lists of mRNAs significantly changing 3-fold or 5-fold upon expression of NKX3.1 were assembled ( Data set 2C).

### RNA isolation and Q-PCR analysis

LH cells were infected with 20 µl of Ad-GFP or Ad-GFP-NKX3.1 viruses and total RNA was isolated after 6, 8, 10, and 12 h using the RNeasy mini kit (Qiagen, Valencia, CA). RNA concentrations were determined by measuring absorption at 260 nm in a spectrophotometer. Aliquots of 2 μg of total RNA from each sample were reverse-transcribed into cDNA using an Omniscript RT kit (Qiagen) according to the manufacturer's instructions. Quantitative Real-Time PCR was performed using Brilliant SYBR Green QPCR Master Mix (Stratagene, La Jolla, CA) and the Mx3000 Real-Time PCR System (Stratagene). Gene specific primers were designed using the Primer3 algorithm ( http://frodo.wi.mit.edu/) as shown below. PCR reactions were run according to the protocol for the Brilliant SYBR Green QPCR Master Mix. Briefly, PCR was carried out using a final concentration of 0.2 μmol of the primer pairs, 50 ng of cDNA template and 12.5 μl of Brilliant ^®^ SYBR Green QPCR Master Mix. The volume was adjusted to 25 μl by adding RNase-free water. The thermocycling protocol began with a 3 min denaturation at 95°C, a 40 cycle amplification program consisting of 30 s denaturation at 95°C, 1 min annealing at 55°C and 30 s extension at 95°C. Upon conversion of raw ct values to linearly related X(0) values, expression values were normalized to GAPDH, and expression changes were expressed as ratios of mRNA levels in NKX3.1 infected versus GFP infected cells (NKX3.1/GFP). The ratios were log2 transformed and averaged across two technical replicates, and standard deviations were calculated.

Primer sequences used for Q-PCR:

HSPA6_F        CCGTGAAGCACGCAGTGAT

HSPA6_R        ACGAGCCGGTTGTCGAAGT

TAGLN_F       GCTGGAGGAGCGACTAGTGG

TAGLN_R       CCTCCTGCAGTTGGCTG

CDH2_F         TGGAACGCAGTGTACAGAATCAG

CDH2_R         TTGACTGAGGCGGGTGCTGAATT

CCND2_F       TACCTTCCGCAGTGCTCCTA

CCND2_R       TCACAGACCTCCAGCATCCA

STAT2_F         CACCAGCTTTACTCGCACAG

STAT2_R         TGGAAGAATAGCATGGTAGCCT

EEF1A2_F       GCTGAAGGAGAAGATTGACC

EEF1A2_R       TTCTCCACGTTCTTGATGAC

CDKN1A_F     TTGTCTTTCCTGGCACTAAC

CDKN1A_R     CCCTCGAGAGGTTTACAGTC

HES1_F           GCATCTGAGCACAGAAAGTC

HES1_R           CTGTCATTTCCAGAATGTCC

S100A2_F       GGGAAATGAAGGAACTTCTG

S100A2_R       CACATGACAGTGATGAGTGC

TNFa_F1        GTGGACCTTAGGCCTTCCTC

TNFa_R1        ATACCCCGGTCTCCCAAATA

TNFa_F2       CCCAGGCAGTCAGATCATCTT

TNFa_R2       TCTCAGCTCCACGCCATT

### Measurement of cell proliferation

LH cells were seeded in 384-well plates at a density of 2000 cells per well. After 24 hours, cells were transduced with Ad-GFP-NKX3.1 or control Ad-GFP adenoviruses for the times indicated in Figure 6D–F. Proliferation (i.e. DNA synthesis) was measured using the Click-iT ^®^ EdU Alexa Fluor ^®^ 594 HCS kit (Invitrogen, Carlsbad, CA) according to the manufacturer's instructions. Briefly, 10 µM 5-ethynyl-2′-deoxyuridine (EdU) was added to culture media for one hour, and cells were fixed with 3.7% formaldehyde, washed with PBS twice, permeabilized with 0.1% Triton X-100 in PBS, stained with Click-iT Alexa Fluor 594 dye, and counterstained with 1 µg/mL Hoechst 33342 (Blue). Plates were scanned and analyzed by using a Celigo automated cytometer at dual wave length to detect Hoechst dye (total cell count) and Alexa Fluor 594 (cells incorporating EdU and thus undergoing DNA synthesis). Four images per well were obtained at each wave length, and the percentage of proliferating cells was calculated by dividing the number of Alexa positive cells by the total cell number.

MAP kinase inhibitors and neutralizing antibodies were added two hours after viral transduction. JNK inhibitors SP600125 (EMD Chemicals Inc, San Diego, CA) and p38 inhibitor SB203580 (Enzo Life Sciences, Farmingdale, NY) were used at 20 µM. Mouse IgG directed against TNFα (Clone *6401,* R&D Systems, Minneapolis, MN) and whole mouse IgG as a control (Jackson ImmunoResearch Laboratories, West Grove, PA) were used at 5 µg/ml.

### Pathway and network analysis

Ingenuity Pathway Analysis (IPA, Ingenuity Systems) was used for pathway and network analysis. The bulk of the analysis was performed with the 5× dataset (mRNAs showing a significant ≥ 5-fold change upon expression of NKX3.1). The 3× dataset was used for the MYC network. Datasets were imported into IPA, and analyzed with the following settings: Reference Set: Ingenuity Knowledge Base (Genes + Endogenous Chemicals); Network Analysis: Direct and Indirect Relationships; Data Source: Ingenuity Expert Findings; Confidence: Experimentally Observed; Species: Mammal (human, mouse, rat) and Uncategorized (e.g. chemicals); Tissue and Cell Lines: All.

### NextBio analysis

The 5× dataset was uploaded to the NextBio server through the Sanford-Burnham portal. 153 of the 158 features of the 5× dataset were recognized and could be interpreted by NextBio. The analysis was performed using default settings. Significantly enriched transcription factor binding sites were identified through corresponding Biogroups. The overlap between the 5× dataset and the gene expression study by Nanni *et al.* ^34^ was identified through a search against all curated studies.

### Indirect immunofluorescence staining

Flag-NKX3.1 transfected LNCaP cells were seeded onto 15 mm poly-lysine coated glass cover slips, and fixed using formaldehyde (3.7% in PBS). Samples were stained with mouse monoclonal FLAG (Sigma) or goat polyclonal NKX3.1 antibodies (Santa Cruz). Alexa Fluor 568 (red) donkey anti-mouse IgG and Alexa Fluor 488 (green) donkey anti-goat IgG conjugate antibodies (Life Technologies Cat# A10037, RRID:AB_11180865 and Cat# A11055, RRID:AB_10564074) were used as secondary antibodies. The nuclei were stained with 4’–6’ diamidino-2-phenylindole (DAPI). Samples were imaged on a Nikon Type 120 inverted fluorescent microscope using 60× magnification.

## Results

### The NKX3.1 interactome

**Figure S1.**
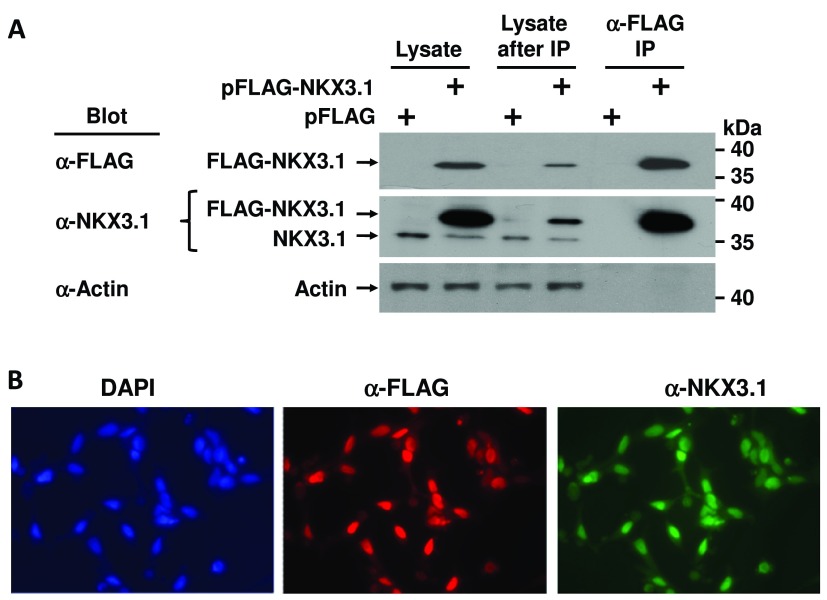
Transfection of FLAG-NKX3.1 expression into LNCaP cells and affinity purification. ( **A**) LNCaP cells were transfected with pFLAG-NKX3.1 plasmid or with the empty pFLAG vector. Total cell lysate (lanes 1 and 2) was absorbed to anti-FLAG resin and eluted with FLAG peptides (lanes 5 and 6). The depleted cell lysate after affinity purification is shown in lanes 3 and 4. Immunoblots were probed with the indicated antibodies. The blot with NKX3.1 shows the overexpressed FLAG-NKX3.1 and the endogenous NKX3.1 protein (middle panel). Actin was used as loading reference. Cropped blot images are shown; see Figure S9 for full images. ( **B**) LNCaP cells were transfected with pFLAG-NKX3.1 plasmid, and FLAG-NKX3.1 was detected by indirect immunofluorescence staining with FLAG or NKX3.1 antibodies.

Reasoning that the NKX3.1 interactome may be most effectively profiled in cells that naturally express this protein, we transiently expressed FLAG epitope-tagged NKX3.1 in LNCaP human prostate cancer cells. FLAG-NKX3.1 was approximately 5-fold in excess over endogenous NKX3.1 ( Supplementary Figure S1A) but localized primarily to cell nuclei ( Supplementary Figure S1B). The proteasome inhibitor MG132 was added 4 hours prior to lysate preparation in order to slow the rapid clearance via the ubiquitin-proteasome pathway to which NKX3.1 is normally subjected ^35, 36^. Cell lysate was absorbed to anti-FLAG M2 resin, and specifically retained proteins were eluted with FLAG peptide. Four independent affinity purifications were performed in parallel with mock purifications of lysate of cells transfected with empty vector. The eluates were examined by SDS-PAGE ( Figure 1A) and subjected to LC-MS/MS analysis in order to determine their protein composition. Altogether, 315 proteins were identified at a false-positive rate of ≤ 0.01 ( Data set 1A).

The protein dataset was subjected to background subtraction and abundance-based filtering to arrive at a list of 58 high confidence NKX3.1 interacting proteins (see Materials and methods and Data set 1B). Fifty five of the 58 proteins were identified in at least two independent purifications, and 27 were identified in at least three purifications ( Figure 1B, Data set 1C). Five proteins were consistently identified as NKX3.1 interaction partners in all four independent purifications, namely NKX3.1, the DNA repair proteins XRCC5/Ku80 and PARP1, and the protein synthesis proteins RPS9 and PABPC1.

We next performed a relative quantification of the NKX3.1 interactome based on spectral counting ^29^. Upon summing the molecular weight adjusted spectrum counts of each protein across the four mock and NKX3.1 purifications, we derived background corrected quantifications by either subtracting summed mock values from summed NKX3.1 bait values (NKX3.1 – Mock) or by dividing NKX3.1 bait values from mock values (NKX3.1/Mock) to obtain the factor by which a protein was enriched in the NKX3.1 bait samples over the mock sample. Both methods confirmed the expectation that NKX3.1 was the most abundant protein identified in the FLAG affinity purifications ( Figure 1C, D). We also performed Reactome Functional Interaction analysis to construct a functional interaction network of NKX3.1 binding proteins derived from manually curated literature data ^32^. The network was clustered into modules and enriched functional pathways/reactions were identified ( Figure 2A).

Among the 10 most abundant co-purifying proteins were the components of the DNA-dependent protein kinase (DNA-PK) holoenzyme, XRCC5/Ku80, XRCC6/Ku70, and poly(ADP) ribose polymerase (PARP1) ( Figure 2A). DNA-PK and PARP1 have important functions in DNA double strand break repair, recombination, and telomere maintenance but are also involved in chromatin and transcriptional control ^37– 39^. For example, Ku proteins associate with a series of homeodomain proteins (HOXC4, OCT1, OCT2, DLX2) thereby recruiting them to DNA ends where they are phosphorylated by DNA-PK ^40^. Such phosphorylation was proposed to lead to DNA damage-dependent changes in their transcriptional activities. ADP-ribosylation mediated by PARP1 can stimulate the ability of DNA-PK to phosphorylate protein substrates ^41^. Our interactome data provide a possible mechanism underlying the previously observed localization of NKX3.1 to sites of DNA damage ^24^, although the functional consequences of these interactions for NKX3.1 transcriptional activity remain to be established. Regardless, follow-up co-immunoprecipitation experiments showed that overexpressed NKX3.1 readily interacted with endogenous XRCC5/Ku80, XRCC6/Ku70, and PARP1 ( Figure 2B). Interaction of DNA-PK with ectopically expressed NKX3.1 was very recently reported in an independent study ^42^. We show here that endogenous NKX3.1 also interacts with XRCC5/Ku80, XRCC6/Ku70, and PARP1 ( Figure 2C).

Among the top ranking NKX3.1 interacting proteins was also interleukin enhancer binding factor 2 (ILF2/NFAT 45 kDa) ( Figure 1D). This protein was previously shown to interact with the DNA-PK-Ku complex ^43^ and to be part of a ribonucleoprotein assembly containing heterogeneous nuclear ribonucleoproteins (hnRNPs), the heat shock protein HSPA8, the poly-A binding protein PABC1, nucleolin (NCL), and several ribosomal proteins ^44^, all of which were also identified here as components of the NKX3.1 interactome ( Figure 1C,D, Data set 1A). Most of these interactions were also represented in the Reactome network ( Figure 2A). Two additional subunits of this particle, ILF3 and YBX1 were also identified, albeit at low levels ( Data set 1A). hnRNPs function in multiple processes, including mRNA splicing, dynamics, stability, and translation, telomere maintenance, DNA repair, and chromatin remodeling and transcription ^45^. They are also major constituents of the nucleolar proteome, which additionally comprises many of the NKX3.1 interacting proteins listed above, including the DNA-PK complex, PARP1, HSPA8, and ribosomal proteins as well as the RNA helicases DDX3 and DDX5 ^46, 47^. Although the significance of these interactions remains unclear, they may reflect a close physical coupling of NKX3.1-dependent mRNA transcription to mRNA processing ^48^ and/or hitherto unappreciated role for NKX3.1 in nucleolar ribosome biogenesis and cytoplasmic mRNA transport. A similar proposition was made to rationalize the interactome of the transcription factor SOX2, which shares remarkable overlap with the NKX3.1 interactome ^49^.

Another highly abundant NKX3.1 interactor is the chromatin and nuclear assembly regulator BANF1 ( Figure 1D). This interaction was confirmed by co-immunoprecipitation ( Figure 2B). BANF1 was previously shown to bind two other proteins identified in the NKX3.1 interactome, emerin (EMD) and thymopoetin (TMPO) ^50^. In addition, BANF1 interacts with several other homeodomain transcription factors and regulates the transcriptional activity of one of them, CRX ^51^. It is thus likely that BANF1, in complex with emerin and thymopoetin, is involved in NKX3.1-mediated gene regulation. The nuclear matrix attachment proteins SAFA/HNRNPU and SAFB, which were also identified as NKX3.1 interacting proteins, may also participate in this process.

Finally, we identified an interaction of NKX3.1 with the homeobox transcription factor HOXB13 ( Data set 1C). This interaction was confirmed by co-immunoprecipitation ( Figure 2A). HOXB13 also interacts with the androgen receptor and regulates the cellular response to androgen ^52^. In addition, germline mutations of HOXB13 significantly increase risk of hereditary prostate cancer through unknown mechanisms ^53^. However, further studies discounted the intriguing possibility that mutation of HOXB13 alters its interaction with NKX3.1 (CCY & DAW, unpublished observation).

### NKX3.1-induced transcriptional program

**Figure 3.**
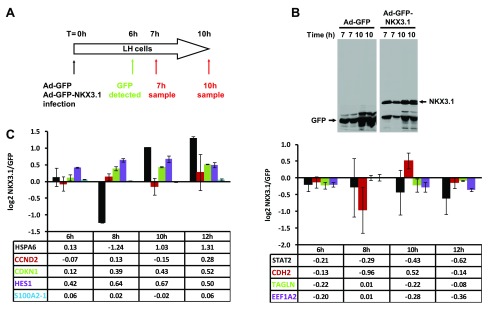
Adenovirus-mediated expression of NKX3.1 in LH prostate epithelial cells regulates specific mRNAs. ( **A**) Schematic representation of the time course of the experiment. LH cells were infected in duplicate with adenoviruses driving the expression of either GFP alone or GFP and NKX3.1 from two separate promoters. GFP expression became first apparent by fluorescence microscopy 6 hours after transfection (data not shown). ( **B**) Duplicate cell lysates were prepared 7 and 10 hours after infection, and examined for the expression of GFP and NKX3.1 by immunoblotting. NKX3.1 expression was already detectable at the earliest time point (7 hours). ( **C**) Quantitative RT-PCR analysis of 9 mRNAs whose expression is changed in response to NKX3.1. LH cells were infected with adenoviruses driving the expression of either GFP alone or GFP and NKX3.1, and mRNA was isolated after the indicated time points (6, 8, 10, 12 hours). The RNA samples were analyzed by Q-PCR, and expression values are shown as log2 transformed ratios of the mRNA level in NKX3.1 infected versus GFP infected cells (NKX3.1/GFP). Error bars indicate standard deviations obtained from two replicate measurements. The left panel shows data for 5 mRNAs that were upregulated by NKX3.1 in the array dataset, whereas the right panel shows data for four mRNAs that were downregulated.

Previous determinations of NKX3.1-dependent gene expression signatures have profiled prostates of mice that developed and aged in the complete absence of NKX3.1 ^16, 19, 20^. These signatures may therefore describe adaptive changes that occur in response to long-term depletion of NKX3.1 in addition to its immediate effects on gene expression. We have therefore chosen to acutely introduce NKX3.1 into immortalized human prostate epithelial cells (LH cells ^25^) that do not express detectable levels of NKX3.1 protein (data not shown). We produced adenoviruses driving the expression of either GFP alone or GFP and NKX3.1 from separate promoters (Ad-GFP and Ad-GFP-NKX3.1 viruses, respectively). LH cells were infected with these viruses according to the scheme in Figure 3A. GFP signal became first detectable by live cell fluorescence microscopy 6 hours after infection (data not shown). We therefore harvested duplicate cultures of cells for immunoblotting 7 and 10 hours after infection and determined that NKX3.1 and GFP were expressed at both time points ( Figure 3B). No cytopathic effects of adenovirus infection were observed within the time frame of the experiment. In parallel, we prepared duplicate RNA samples of the 7 hours and 10 hours time points for transcriptome analysis.

**Figure S2.**
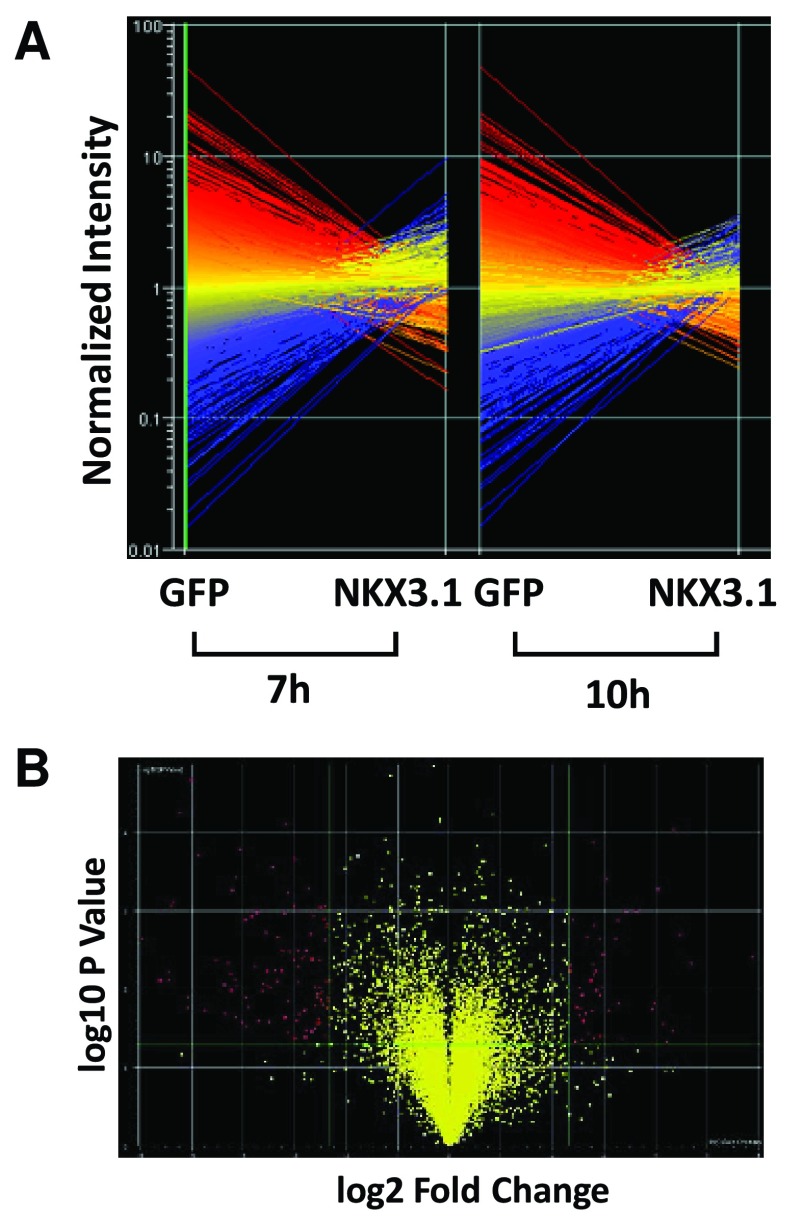
Global gene expression signature of NKX3.1 expression in LH cells. ( **A**) Differential gene expression 7 and 10 h after NKX3.1 expression in LH cells. Note the overall similarity of gene expression differences between GFP and NKX3.1 expressing LH cells at both time points (7 h and 10 h). ( **B**) "Volcano Plot" of differentially expressed genes at the 7 h time point. Features marked in red differed significantly 5-fold between GFP and NKX3.1 expressing samples.

The global changes in transcript levels noted in response to NKX3.1 expression were very similar at the 7 hours or 10 hours time points ( Supplementary Figure S2A). Statistically significant changes were observed for several hundred mRNAs. To reduce the number of mRNA changes to be further interrogated to a manageable number, we arbitrarily set a cut-off of 5-fold change. This yielded lists of 158 differentially expressed genes for the 7 hours time point ( Supplementary Figure S2B) and 165 for the 10 hours time point. Since there was a considerable overlap of both lists, we limited the further analysis to the 7 hours sample. Data sets 2A and B summarize all mRNA expression data. Supplementary Table 1 presents a ranked list of all 107 mRNAs with > 5-fold upregulation, whereas Supplementary Table 2 presents a corresponding list of all 51 mRNAs with > 5-fold downregulation in NKX3.1 expressing LH cells (see also Data set 2C). We chose 5 upregulated and 5 downregulated mRNAs for validation by Q-PCR with a fresh set of replicate RNA samples prepared from cells infected with Ad-GFP or Ad-GFP-NKX3.1 for increasing periods of time. Nine out of the 10 expression changes confirmed the tendency seen from microarrays, although variability was substantial for some measurements ( Figure 3C). We failed to confirm the induction of KRT17 mRNA apparent from the array data (not shown). Additional validation by Q-PCR and immunoblotting is shown in various sections below (see Figure 6).

Examination of the lists of mRNA changes revealed a fundamental reprogramming of gene expression in LH cells upon acute expression of NKX3.1. Overall, the changes were indicative of inhibition of cell proliferation and induction of cell differentiation. For example, 9 epithelial differentiation markers (cytokeratins 5, 6B, 7, 8, 17, 18, 19, stratifin, kallikrein 5) were strongly induced. In addition, the Notch pathway, which is often downregulated in prostate cancers ^54^, was induced (DLL1, HES1, JAG2). The cyclin-dependent kinase inhibitor p21 (CDKN1A), which inhibits cell cycle progression and induces cell differentiation ^55^, was also increased.

Reassuringly, many of the strongest NKX3.1-induced mRNAs encode proteins that were previously shown to be downregulated in human prostate cancer based on immunohistochemistry ( Supplementary Table 1). This included, for example, the calcium binding proteins S100A2 and A14 ^56^, the 14-3-3 protein stratifin ^57, 58^, laminin A ^59^, claudin 7 ^60^, prostasin ^61^, P cadherin ^62^, and kallikrein 5 ^63^. Cyclin D2 is considered an activator of cell cycle progression but was induced by NKX3.1. Remarkably, however, cyclin D2 is typically downregulated in human prostate cancers ^64^. Four mRNAs encoding HSP70s were upregulated ( Supplementary Table 1). HSP70 expression is frequently lost in aggressive prostate cancers ^65^ and experimental HSP70 overexpression inhibits the tumorigenicity of prostate cancer xenografts in mice ^66^. Likewise, three genes encoding the HSP70 co-chaperones DnaJ/HSP40 were upregulated > 5-fold. Lastly, two glutathione transferases were upregulated by NKX3.1, a finding that is consistent with the previous demonstration that NKX3.1 upregulates oxidative stress defense ^20^.

The list of downregulated genes ( Supplementary Table 2) included genes involved in cell migration (actin/myosin-related, collagens 1A1, 5A1, 5A2), several growth factors, and the interferon/STAT pathway. Many of the most downregulated genes were previously shown to be overexpressed in prostate and other cancers ( Supplementary Table 2). This applies, for example, to eukaryotic translation elongation factor 1 alpha (EEF1A2) which is a potential oncogene ^67^, the BMP antagonist gremlin 1 ^68^, and the transcription factor FOXD1 ^69^. N-cadherin, which is frequently found to replace epithelial cadherin forms in prostate cancers (“cadherin switch”) was also strongly downregulated ^70^. Significantly, NKX3.1 also upregulated P cadherin thus reversing the cadherin switch.

We also compared our list of 357 mRNAs that were changed ≥ 3-fold by NKX3.1 with a recent list of 282 mouse genes thought to be direct NKX3.1 targets based on a combination of expression and ChIP-seq data ^16^. Despite the species difference and the diametrical strategies (overexpression versus knockout), 10 genes were represented on both lists ( Supplementary Table 3). This overlap is highly significant when considering that 8 out of these 10 genes were regulated by NKX3.1 in the same direction.

### Pathway analysis

To assess functional modules and signaling pathways affected by NKX3.1, we performed a global analysis with the Ingenuity Pathway Analysis (IPA) package. The analysis was performed with the dataset of mRNAs changing more than 5-fold (“5× dataset”) or, where indicated, with a larger dataset of mRNAs changing more than 3-fold (“3× dataset”, 357 genes). Since identical top scoring pathways were obtained with both datasets, the analysis was largely restricted to the smaller 5× dataset, unless otherwise noted.

**Figure 4.**
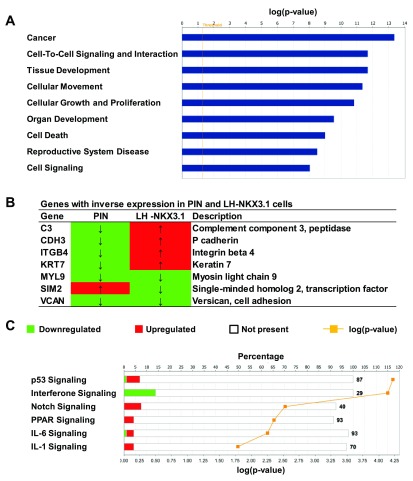
Functions and pathways that are overrepresented in the NKX3.1 gene expression program. ( **A**) Select IPA “Functions” significantly overrepresented in the 5× mRNA set. ( **B**) List of mRNAs with inverse expression in prostatic intraepithelial neoplasia (PIN; ^72^) and NKX3.1 expressing LH cells. mRNAs shown in red are upregulated whereas those shown in green are downregulated. ( **C**) Select IPA “Canonical Pathways” overrepresented in the 5× dataset. The abscissa on the top indicates the percent fraction of all possible pathway components that were represented in the dataset. Since this dataset only contained a relatively small number of 158 mRNAs, a small percent wise overrepresentation of pathway components is statistically highly significant (p < 0.05, see yellow graph).

Consistent with the involvement of NKX3.1 in prostate development, we found highly significant overrepresentation of IPA “Functions” pertaining to development, cell movement, proliferation and cell growth ( Figure 4A). Of particular interest was the term “Reproductive Systems Disease”, which included the subgroup “Prostatic intraepithelial neoplasia” (PIN). PIN is the earliest known precursor lesion of prostate cancer, and frequently shows decreased NKX3.1 levels ^71^. The “PIN” Function contained the seven genes listed in Figure 4B. A previous study determined that six of these genes were downregulated in PIN versus normal prostate, whereas one was upregulated ^72^. Remarkably, five out of the seven genes displayed a mirror image of the changes occurring in PIN when examined in NKX3.1-expressing LH cells ( Figure 4B). These findings suggest that changes in gene expression in early PIN may be causally linked to loss of NKX3.1.

As shown in Figure 4C, a number of pathways were overrepresented that were not readily apparent from the manual curation of the gene lists presented above. For example, the analysis indicated upregulation by NKX3.1 of the p53 and IL1 pathways, in addition to the Notch signaling pathway. Interferon signaling, in turn, appeared to be switched off by acute NKX3.1 expression.

### Network analysis

**Figure 5.**
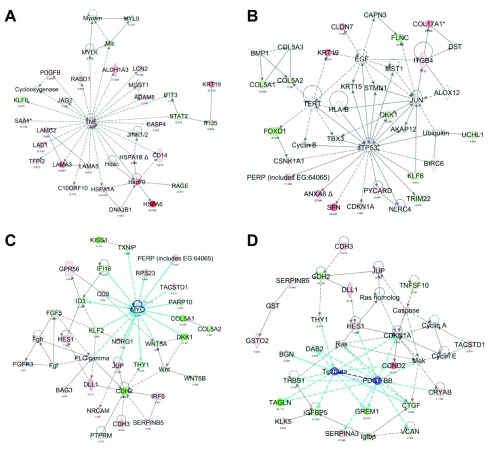
IPA network analysis of the NKX3.1 transcriptional program. ( **A**) TNFα network. Node colors represent the level of up- (red) or down- (green) regulation upon expression of NKX3.1. ( **B**) Tumor suppressor p53 network. The p53-TERT-EGF-JUN quadrangle is highlighted by dark blue edges. ( **C**) MYC network. First degree edges of MYC are highlighted in light blue. ( **D**) PDGFB/TGFβ network. First degree edges are highlighted in light blue, the PDFGB-TGFβ link in dark blue.

**Figure 6.**
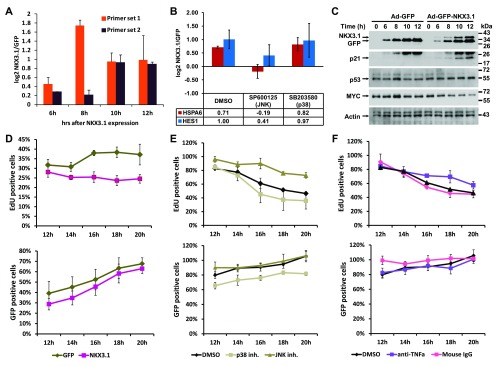
NKX3.1-induced changes in gene and protein expression. ( **A**) Quantitative RT-PCR analysis of TNFα mRNA. LH cells were infected with adenoviruses driving the expression of either GFP alone or GFP and NKX3.1, and mRNA was isolated after the indicated time points (6, 8, 10, 12 hours). The RNA samples were analyzed by Q-PCR with two different primer sets amplifying TNFα mRNA, and expression values are shown as log2 transformed ratios of the mRNA level in NKX3.1 infected versus GFP infected cells (NKX3.1/GFP). Error bars indicate standard deviations obtained from two replicate measurements. ( **B**) LH cells were infected with adenoviruses driving the expression of either GFP alone or GFP and NKX3.1. After 4 hours, 10 μM of the JNK inhibitor SP600125 or 10 μM of the p38 kinase inhibitor SB203580 were added followed by mRNA isolation after 6 hours. The levels of HSPA6 and HES1 were analyzed by Q-PCR. Expression values are shown as log2 transformed ratios of the mRNA level in NKX3.1 infected versus GFP infected cells (NKX3.1/GFP). Error bars indicate standard deviations obtained from two replicate measurements. ( **C**) LH cells were infected with adenoviruses driving the expression of either GFP alone or GFP and NKX3.1, and protein lysates were prepared after the indicated time points (6, 8, 10, 12 hours). The expression of the indicated proteins was determined by immunoblotting. Cropped blot images are shown; see Figure S8. for full images. ( **D**) LH cells were infected with Ad-GFP and Ad-GFP-NKX3.1 viruses, and the rate of DNA synthesis was measured by EdU incorporation after the indicated times (top graphs). The percentage of GFP positive cells was determined as a measure of infection efficiency (bottom graphs). ( **E**) LH cells were infected with Ad-GFP-NKX3.1 virus, and the effect of JNK inhibitor (SP600125, 20 μM) or p38 kinase inhibitor (SB203580, 20 μM) on NKX3.1-mediated suppression of DNA synthesis was measured by EdU incorporation. The percentage of GFP positive cells was determined as a measure of infection efficiency (bottom graphs). ( **F**) LH cells were infected with Ad-GFP-NKX3.1 virus, and the effect of neutralizing antibodies to TNFα or control IgG on NKX3.1-mediated suppression of DNA synthesis was measured by EdU incorporation. The percentage of GFP positive cells was determined as a measure of infection efficiency (bottom graphs).

***TNFα network.*** To obtain a better understanding of the regulatory circuitry underlying NKX3.1-induced modulation of particular functional pathways, we performed network analysis using Ingenuity IPA software. The highest ranking network presented in Figure 5A featured TNFα, a gene that was induced by NKX3.1 ( Supplementary Table 1, Figure 6A), in the center with edges reaching to 27 distinct nodes. Eighteen of these edges were defined by a gene regulatory relationship (i.e. expression edge) thus signifying genes that are known to be either induced or suppressed by TNFα signaling. Further annotation of the TNFα network also connected TNFα to NKX3.1-induced suppression of cell movement through downregulation of action-myosin based mobility components and enhancement of cell adhesion through upregulation of laminins ( Figure 5A). Both processes are considered bona fide hallmarks of tumor suppression. Close examination of every TNFα expression edge revealed considerable concordance between the definition of the edge (based on the published literature) and the actual expression of the target node in response to NKX3.1. Fourteen first degree nodes predicted to be activated by TNFα were also upregulated by NKX3.1 ( Supplementary Table 4). Consistent with MAP kinase signaling being a major downstream pathway activated by TNFα, we found that a chemical inhibitor of JNK but not p38 could partially antagonize NKX3.1-induced expression of HSPA6 and HES1 ( Figure 6B).

**Figure S3.**
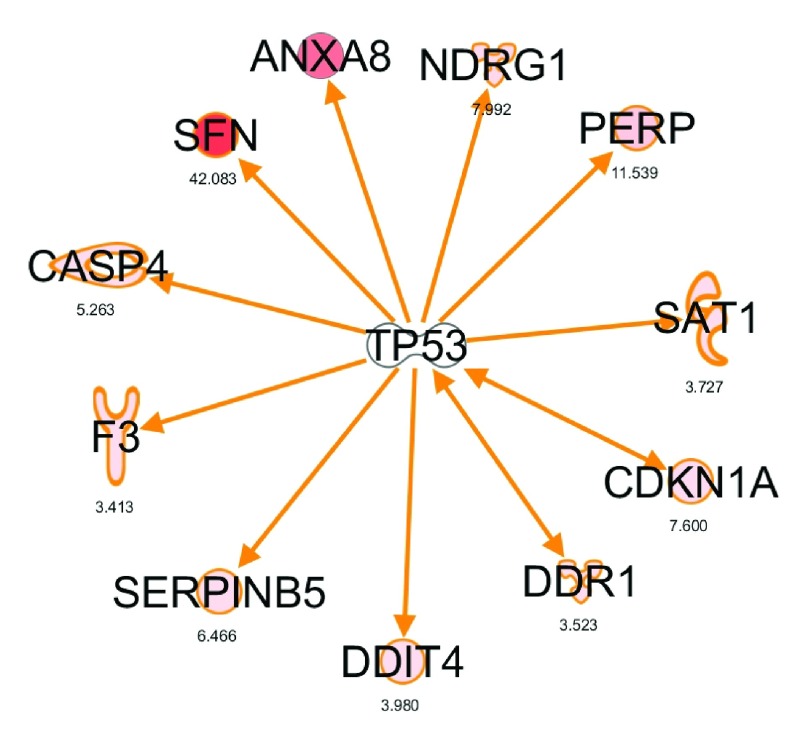
p53-linked expression changes. IPA-based rendering of mRNAs contained in the 5× datasets that were previously shown to be regulated by p53.

***p53 network.*** Another high scoring network featured the tumor suppressor p53 at the center with first degree edges to 8 nodes. Although p53 was upregulated neither at the mRNA nor protein level ( Figure 6C), a finding which is consistent with the well-established activation of p53 at the post-translational level, the network indicated robust induction of some of its known target genes. As shown in Figure 5B, this included the 14-3-3 sigma protein stratifin (SFN), an epithelial differentiation marker missing from many prostate cancers ^57, 73^, the cyclin-dependent kinase inhibitor p21 (CDKN1A, ^74^), and the p53 apoptosis effecter PERP ^75^. Induction of p21 protein by NKX3.1 was confirmed by immunoblotting ( Figure 6C). Annexin A8 (ANXA8) is also known to be upregulated by p53 ^76^. Using the 3× dataset, we pinpointed an additional 7 mRNAs that are upregulated by NKX3.1 as known targets of p53 ( Supplementary Figure S3). These findings suggested that the p53 tumor suppressor pathway is activated by acute induction of NKX3.1 in LH cells. The network contained three additional highly connected nodes, telomerase (TERT), EGF, and JUN, which formed a quadrangle with p53. Although JUN mRNA was not induced by NKX3.1, a positive effect of p53 on JUN was reported previously ^77^.

***MYC network.*** A further high scoring network that was obtained with the 3× dataset was organized around the MYC oncogene ( Figure 5C). MYC itself was 4-fold downregulated by NKX3.1 expression, an effect that was validated by immunoblotting ( Figure 6C). This coincided with downregulation of several genes that were previously found to require MYC function for their expression (TXNIP, IFI16 ^78^). In addition, the MYC interaction partner PARP10 was downregulated upon expression of NKX3.1. Conversely, two genes that are negatively regulated by MYC were activated upon NKX3.1 expression (PERP ^79^, NDRG ^80^), suggesting that NKX3.1-induced downregulation of MYC relieves its repressive effect on these genes. In aggregate, these findings suggest that restoration of NKX3.1 expression in LH cells led to downregulation of pathways normally turned on by MYC. This may contribute to a block in proliferation and promote cell differentiation by NKX3.1. Antagonism of NKX3.1 and MYC in target gene regulation and prostate tumorigenesis was recently also demonstrated in a mouse model ^16^.

**Figure S4.**
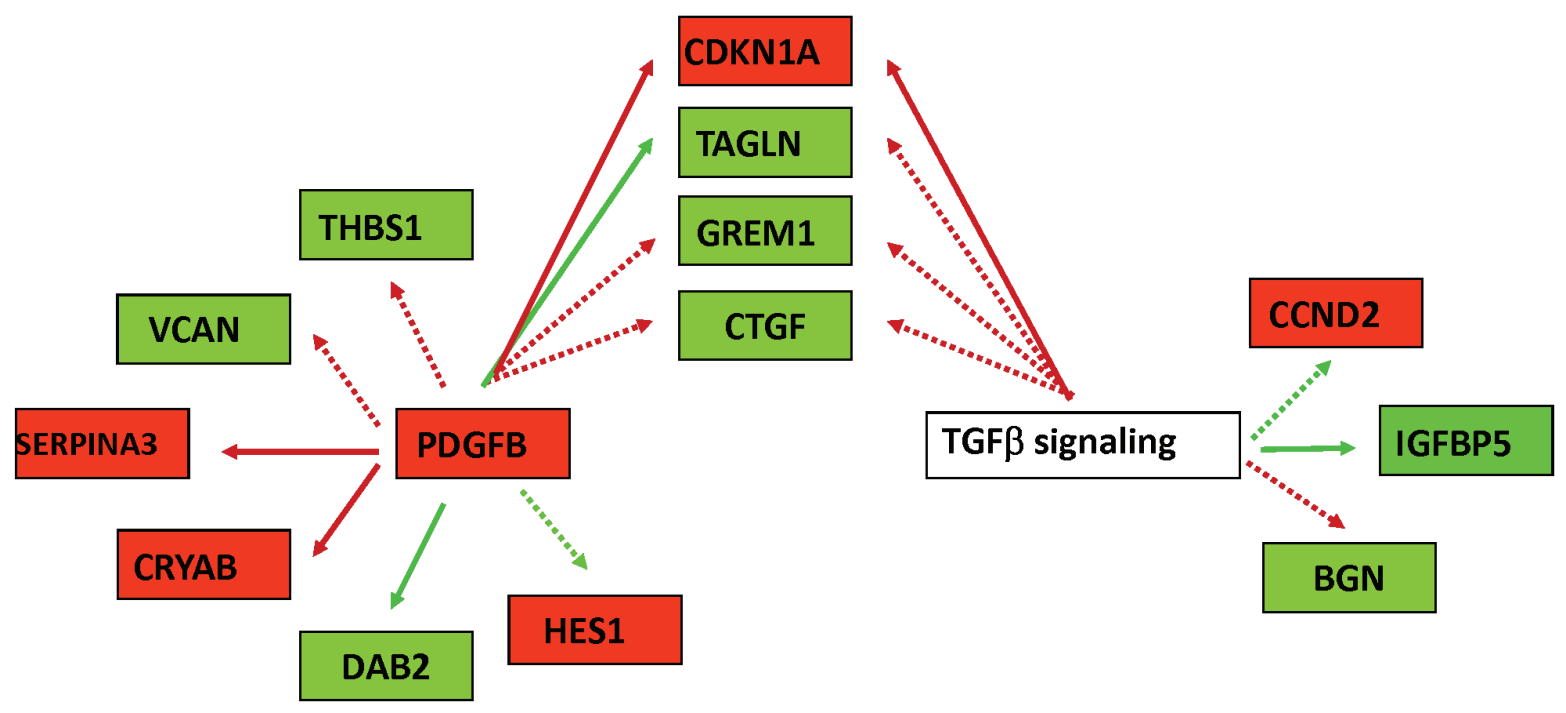
Schematic depiction of PDGFB and TGFβ expression edges according to IPA and comparison with the actual behavior of first degree nodes in response to NKX3.1 expression in LH cells. Green color indicates upregulation, whereas red color signifies downregulation. The arrows represent the expression edges. Solid arrows indicate agreement between observed expression behavior and the behavior expected in response to activation of PDFGB or TGFβ according to the information contained in the IPA database. The stippled arrows indicate disagreement. Example: PDGF is expected to upregulate HES1. Induction of PDGF by NKX3.1 is therefore consistent with the change in HES1 mRNA (edge is solid red arrow). PDGFB is also expected to upregulate THBS1 (red edge), but NKX3.1 expression leads to suppression of THBS1. Hence the edge is a stippled arrow.

***PDGFB/TGFβ network.*** Another network featured PDGFβ (PDGFB and PDFGBB), which was induced 5.1-fold by NKX3.1. The induction of PDGFB mRNA and the expression of many of its first degree interacting nodes, is consistent with PDGFB signaling being upregulated by NKX3.1. For example, three nodes that were upregulated by NKX3.1 (CRYAB, SERPINA3, CDKN1A) and two nodes that were downregulated (DAB2, TAGLN) were previously shown to be controlled by PDGFB in the same manner ( Supplementary Figure S4; ^81, 82^). PDGFB is also known to activate PPAR/RXRα-dependent transcription. Notably, RXRα is itself upregulated by NKX3.1 (5.7-fold), hence explaining the overrepresentation of PPAR signaling in the canonical pathway analysis above ( Figure 4C). Since PPAR signaling is known to suppress prostate cancer cell proliferation ^83^, it may be relevant to NKX3.1-mediated tumor suppression.

PDGFB shares a number of nodes with another growth factor, TGFβ ( Figure 5D). Although TGFβ1 mRNA was not altered by NKX3.1, the more abundantly expressed TGFβ2 was downregulated ( Supplementary Table 5). Most first-degree nodes emanating from TGFβ were downregulated by NKX3.1 expression ( Supplementary Figure 3). An additional 25 genes in the TGFβ signaling pathway were either downregulated or unchanged by NKX3.1, further suggesting that NKX3.1 does not activate TGFβ signaling ( Supplementary Table 5). Since TGFβ is a strong driver of the epithelial-to-mesenchymal transition (EMT, ^84^), NKX3.1-mediated suppression of TGFβ signaling may contribute to its differentiation-inducing activity.

### Network connectivity

**Figure 7.**
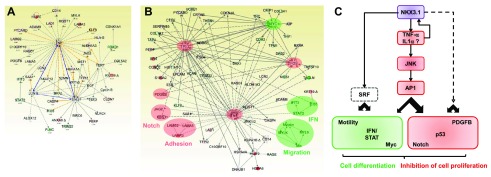
Framework of the NKX3.1 transcriptional program. ( **A**) The merged TNFα-p53 network. Network links are highlighted in yellow. Direct edges between TNFα, p53, and JUN are emphasized in blue color. ( **B**) Construction of a network containing the major factors implicated in the NKX3.1 transcriptional program, including FOS/AP1, MYC, and p53. Modules activated by NKX3.1 expression are shaded in red and those suppressed in green. ( **C**) Tentative framework of NKX3.1-dependent changes to cellular modules. Based on the induction of TNFα and FOS mRNA by NKX3.1, and the antagonistic effects of JNK inhibitors on NKX3.1-mediated gene expression and cell proliferation, the framework proposes that TNFα signaling results in activation of AP1 and modulation of downstream genes and functional modules (red squares symbolize upregulation/activation, green squares downregulation). Additional pathways (stippled lines) may impinge on SRF and other transcription factors (not shown).

In an attempt to obtain a more cohesive view of the global effects of NKX3.1 on prostate gene expression, we merged individual networks. For simplicity, only expression edges were included in Figure 7A. Not only were TNFα and p53 directly linked through an expression-based edge, but several of their individual first degree nodes were targets of edges emanating from both TNFα and p53. For example, TFP12 and CASP4 are positively regulated by both TNFα and by p53 ^77, 85– 87^.

The AP1 transcription factor subunit JUN, which was part of the p53 network ( Figure 5B) was linked to TNFα resulting in a triangular configuration ( Figure 7A). Whereas both TNFα and p53 are known to stimulate the expression of JUN and AP1 activity ^77, 88^, NKX3.1 expression did not significantly affect the mRNA level of cJUN (-1.21-fold change) or JUND (+1.25-fold change). However, the JUN interaction partner FOS was increased 3.9-fold by NKX3.1. Since FOS maintains exactly the same edges within the network as JUN (data not shown), AP1 transcriptional activity appears to be upregulated in response to NKX3.1 expression.

Finally, we manually integrated the TNFα network with the connections to all major factors the network analysis had implicated in the NKX3.1 transcriptional program, including FOS/AP1, MYC, and p53. Despite the complexity of the resulting network, a tentative framework for NKX3.1-induced transcriptomic changes is becoming readily apparent ( Figure 7B, C). According to this framework, NKX3.1 expression in LH cells results in the activation of the TNFα pathway. This in turn leads to activation of the p53, Notch, PDGFB, and AP1 pathways. Conversely, the MYC and interferon/STAT pathways are turned off. Through Q-PCR and immunoblotting, we have already confirmed several of these predictions (see Figure 3C for p53, Notch, PDGFB, STAT, and Figure 6 for TNFα, MYC, and p53). In addition, transduction with NKX3.1 expressing virus led to growth inhibition of LH cells relative to virus expressing GFP alone ( Figure 6D). Notably, growth inhibition was partially rescued by JNK inhibitor and by a neutralizing antibody against TNFα ( Figure 6E, F). These observations further support a role of NKX3.1 in inducing a block to cell division and promoting cell differentiation via a TNFα/JNK/AP1-dependent pathways.

### Enrichment of transcription factor binding sites

**Figure S5.**
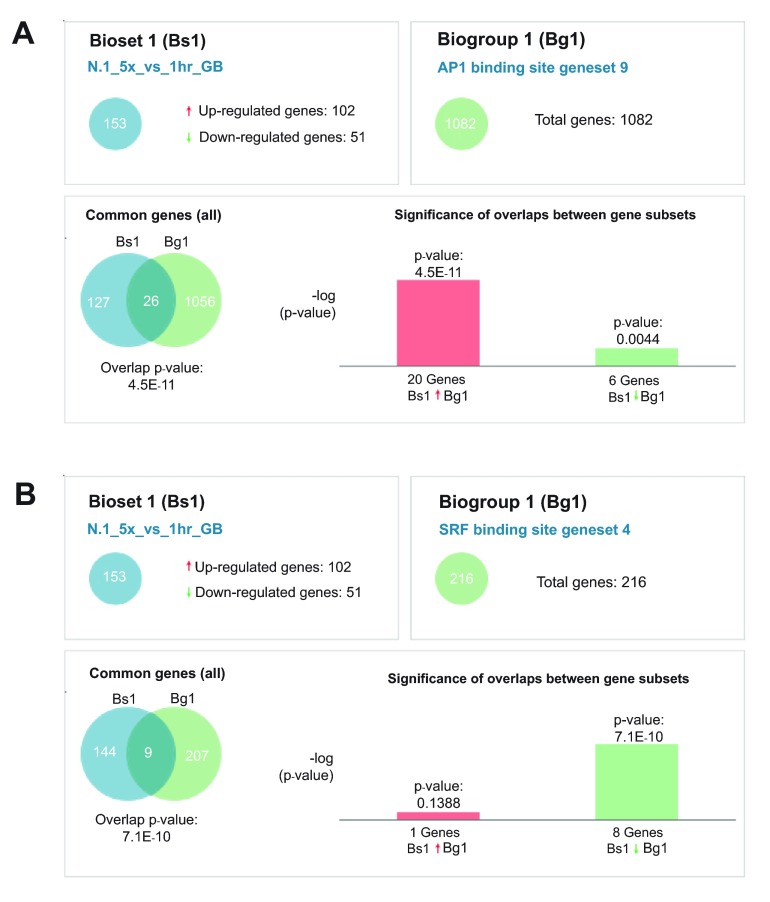
Enrichment of putative conserved AP1 and SRF binding sites in genes affected by NKX3.1 expression in LH cells as determined with NextBio. ( **A**) The top panels summarize the datsets: Bioset 1 = 5× dataset of mRNAs affected by NKX3.1 expression; Biogroup 1 = AP1 binding site gene set according to ^1^. The bottom panel illustrates the overlap between Bioset 1 and Biogroup 1 in a Venn diagram (left) and in bar graphs (right). The bar graph shows that most genes containing conserved AP1 binding sites are activated by NKX3.1 expression. The individual genes are indicated in Supplementary Table 1 and Supplementary Table 2. ( **B**) Same as above for serum response factor (SRF).

We next employed the NextBio platform to relate our expression data to previously published large-scale genomics data. One dataset that matched with high statistical significance (p = 4.5E-11) featured a set of 1082 genes containing evolutionarily conserved genomic binding sites for AP1 ^89^. Twenty six of these genes were represented in our list of ~150 NKX3.1 responsive genes with 20 being induced by NKX3.1 ( Supplementary Table 1, Supplementary Table 2, Supplementary Figure 5A, Data set 2D). Combined with the evidence from network analysis and the upregulation of FOS, these findings suggest that NKX3.1 causes AP1 activation and/or cooperates with AP1 in gene activation. Consistent with this conjecture is the well-known induction of JUN N-terminal kinase (JNK) activity by TNFα signaling, which enhances the transcriptional activity of JUN. Finally, NFκB which is also induced by TNFα signaling, can cooperate with AP1 at some promoters ^90^.

A second DNA binding motif that was overrepresented (p = 1.6E ^-5^) in NKX3.1 responsive genes conforms to a binding site for serum response factor (SRF). 216 human genes contain the serum response element (SRE) motif in a promoter proximal context that is conserved in mouse, rat, and dog ^89^. These 216 genes included 9 genes that were represented on our dataset, all but one of which was suppressed by NKX3.1 ( Supplementary Table 2, Supplementary Figure 5B, Data set 2E). Since NKX3.1 is known to physically interact with SRF ^17^, our data strongly suggests that NKX3.1 cooperates with SRF in transcriptional suppression.

### Comparison with human prostate cancer data

Nextbio analysis also revealed a highly significant match with a study comparing gene expression in human prostate cancer tissues ^34^. This study profiled 22 cell lines derived from surgical samples of prostate cancer patients with clinically localized disease and absence of hormonal neo-adjuvant treatment before surgery. In keeping with these selection criteria for early cancers, the cell lines (and primary tumors they were derived from) had suffered loss of 8p21 (i.e. NKX3.1) but did not display genetic abnormalities typical of more advanced prostate cancers (e.g. loss of PTEN, amplification of MYC and androgen receptor). 3415 mRNAs were significantly changed in prostate cancer cell lines relative to normal prostate.

**Figure S6.**
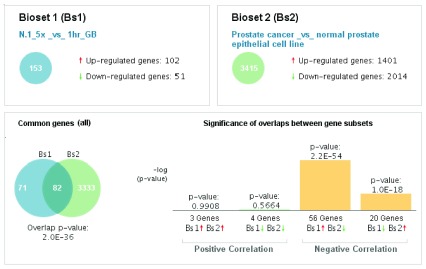
Overlap in mRNA expression between the 5× dataset and in human prostate cancer derived cell lines. ( **A**) The top panels summarize the datasets: Bioset 1 = 5× dataset of mRNAs affected by NKX3.1 expression; Bioset 2 = Prostate cancer derived cell lines versus normal prostate epithelial cells ^2^. The bottom panel illustrates the overlap between Bioset 1 and Bioset 2 in a Venn diagram (left) and in bar graphs (right). The bar graph highlights the largely opposite gene expression patterns in the two biosets. The individual genes are indicated in Supplementary Table 1 and Supplementary Table 2.

Of 153 differentially expressed genes in our dataset, 82 (53%) were also changed in prostate cancer derived cell lines (PCaDCL), a highly significant overlap (p = 2.0E ^-36^, Supplementary Figure 6; Data set 2F). Of the 82 overlapping genes, 60 were downregulated and 22 were upregulated in PCaDCL versus PrEC. Strikingly, 93% of the mRNAs downregulated in PCaDCL were induced by expression of NKX3.1 in LH cells ( Supplementary Table 1). In addition, 19 of the 20 genes upregulated in PCaDCL were downregulated by NKX3.1 ( Supplementary Table 2). Moreover, many of the mRNA expression changes observed in the PCaDCL microarray study were independently confirmed at the protein level by immunohistochemistry of prostate cancer tissue samples ( Supplementary Table 1 and Supplementary Table 2). These analyses strongly suggest that the principal gene regulatory networks that are affected by NKX3.1 expression in LH cells are inversely perturbed in early human prostate cancer marked by loss of this tumor suppressor.

NKX3.1 expression and interactions Dataset Data set 1: Summary of NKX3.1 interacting proteins
Data set 1A: All proteins identified in four independent mock and FLAG-NKX3.1 affinity purifications Data set 1B: List of 58 high confidence NKX3.1 interacting proteins Data set 1C: Lists of proteins shown in the Venn diagram in Fig. 1B
Data set 2: Summary of NKX3.1-regulated gene expression
Data set 2A: All raw mRNA expression data Data set 2B: Averaged mRNA expression data Data set 2C: List of mRNAs that show a greater than 5-fold change (p greater or even to 0.05) in cells expressing NKX3.1 for 7 hours (= "5x data set") Data set 2D: list of NKX3.1 regulated genes containing conserved AP1 binding sites Data set 2E: List of NKX3.1 regulated genes containing conserved SRF binding sites Data set 2F: List of NKX3.1 regulated mRNAs that are inversely regulated in human prostate cancer derived cell lines Click here for additional data file. Copyright: © 2014 Yang CC et al. 2014 Data associated with the article are available under the terms of the Creative Commons Zero "No rights reserved" data waiver (CC0 1.0 Public domain dedication).

## Discussion

We have employed a series of global approaches to explore the tumor suppressor function of NKX3.1. The NKX3.1 interactome revealed a complex pattern of interactions with DNA repair proteins and with other transcriptional regulators such as ILF2 and BANF1 that predict a similarly complex transcriptional program enacted by NKX3.1. Indeed, global analysis of the gene expression pattern actuated by acute expression of NKX3.1 in immortalized human prostate epithelial cells with a basal phenotype (LH cells ^25, 91^) revealed a rapid and extensive re-programming with 158 mRNAs changing ≥ 5-fold and 331 mRNAs changing ≥ 3-fold. This complex pattern was interrogated by network analysis to account for the recognition that representation of cellular processes and reactions as linear pathways is often an oversimplification that does not accurately reflect the complexity of intracellular wiring ^92^.

Network analysis indicated NKX3.1-dependent modulation of a series of interconnected functional modules and enabled a tentative framework for the transcriptional program induced by NKX3.1 in human prostate epithelial cells. Broadly speaking, NKX3.1 activation culminates in the downregulation of cellular motility as well as MYC and IFN/STAT activity and in the upregulation of p53 activity, the Notch pathway, and PDGF signaling ( Figure 7C). Many of these changes are readily consistent with the tumor suppressor function of NKX3.1 observed in knockout mice ^3– 5^.

Importantly, network analysis allowed us to pinpoint several unanticipated pathways on which NKX3.1 appears to impinge. For example, the analysis suggested a major role for TNFα whose mRNA was induced by NKX3.1. TNFα is a well-established inducer of MAP kinase signaling, including JNK and p38 kinases. Significantly, IL1α was also induced by NKX3.1 ( Supplementary Table 1) thus further augmenting MAPK activation. JNK activates AP1 transcriptional activity thus readily rationalizing the strong overrepresentation of AP1 binding sites in NKX3.1 responsive genes. Localized NKX3.1-mediated TNFα-JNK signaling in prostate epithelial cells may promote and maintain their differentiation state thus suppressing tumorigenesis. The important role of JNK signaling in cell differentiation is well established ^93, 94^. The finding that pro-inflammatory cytokines also destabilize NKX3.1 protein ^36^ indicates a negative feedback loop that may counteract their pro-apoptotic function ( Figure 7C).

Importantly, the NKX3.1-induced gene signature is, to a large extent, a mirror image of the gene expression pattern found in early human prostate cancers devoid of NKX3.1 ^34^. This inverse pattern further suggests that NKX3.1 is a key driver of luminal cell differentiation, whereas loss of NKX3.1 would allow luminal cells to dedifferentiate into a state with higher proliferative capacity thus making them more vulnerable to the acquisition of additional oncogenic events perhaps augmented by concurrent defects in DNA repair. Clearly such additional events are essential for prostate carcinogenesis given that PIN in NKX3.1 knockout mice does not progresses to overt prostate cancer, unless further genetic changes are incurred ^5– 8^.

## Data availability

The data referenced by this article are under copyright with the following copyright statement: Copyright: © 2014 Yang CC et al.

Data associated with the article are available under the terms of the Creative Commons Zero "No rights reserved" data waiver (CC0 1.0 Public domain dedication). 

*figshare:* NKX3.1 expression and interactions Dataset. Doi: 10.6084/m9.figshare.1002064 ^95^

## Figures and Tables

**Figure S7.**
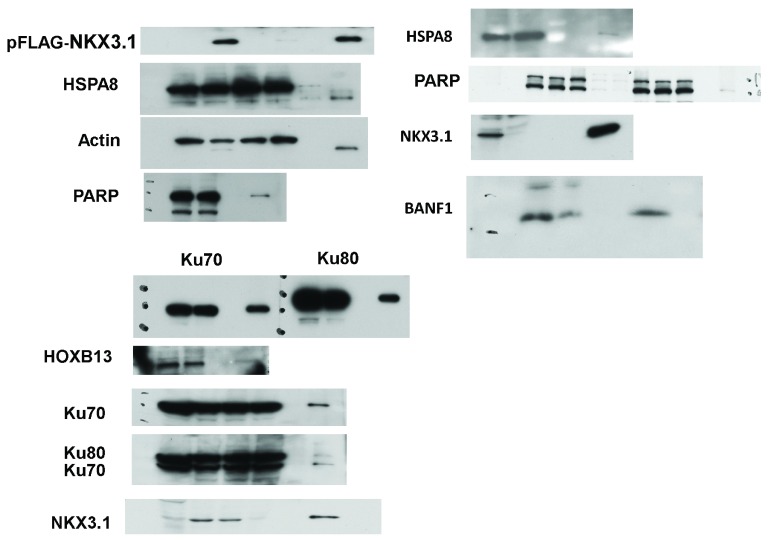
Uncropped immunoblots for Figure 2.

**Figure S8.**
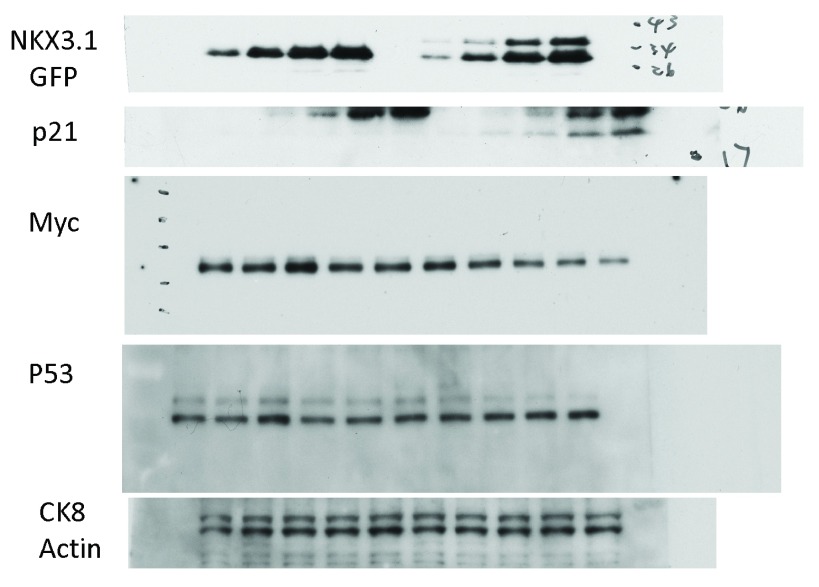
Uncropped immunoblots for Figure 6.

**Figure S9.**
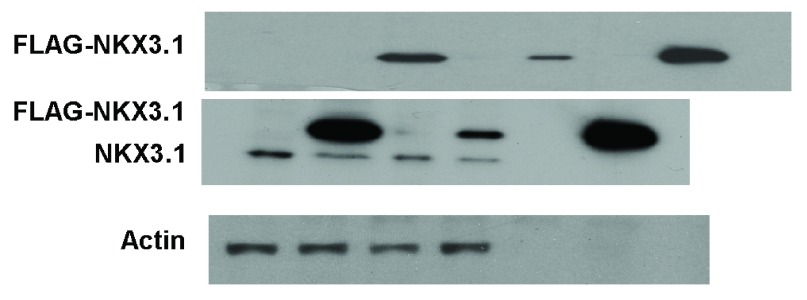
Uncropped immunoblots for Figure S1.

**Figure:**
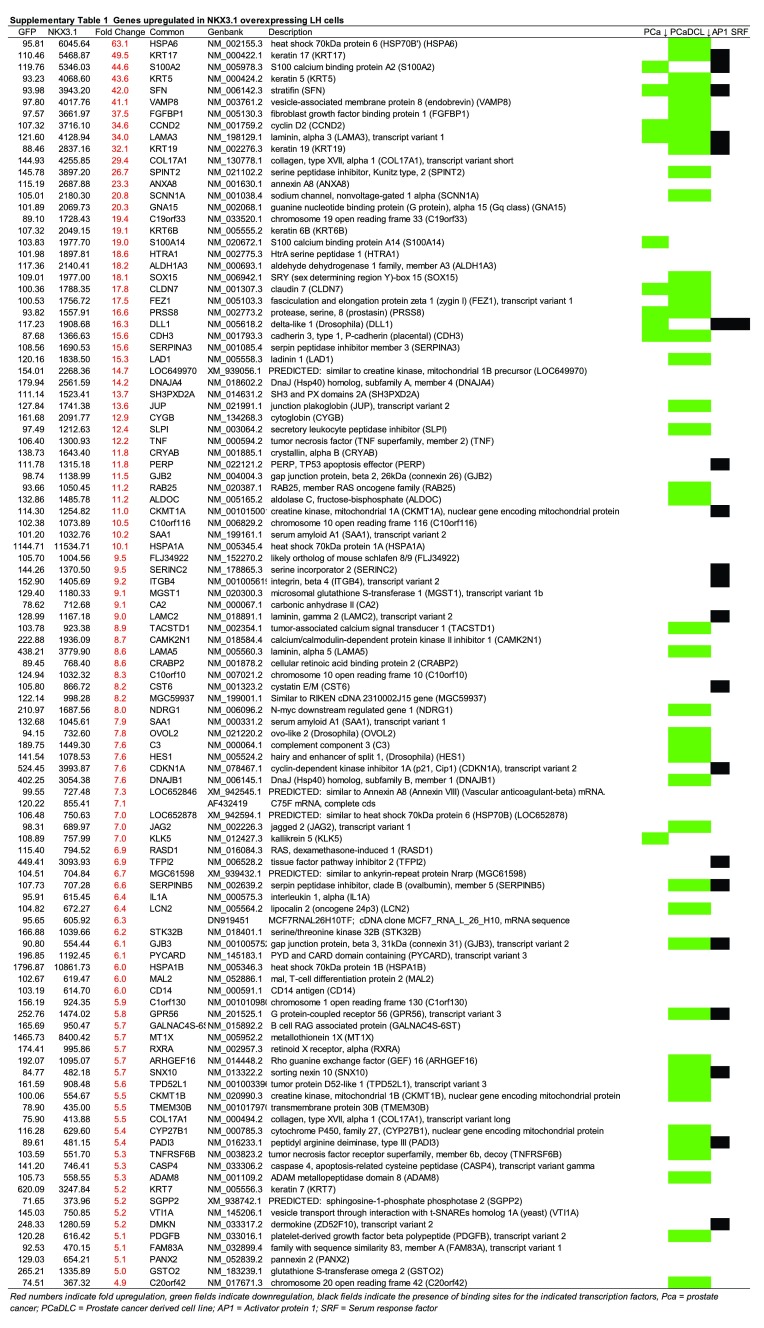


**Figure:**
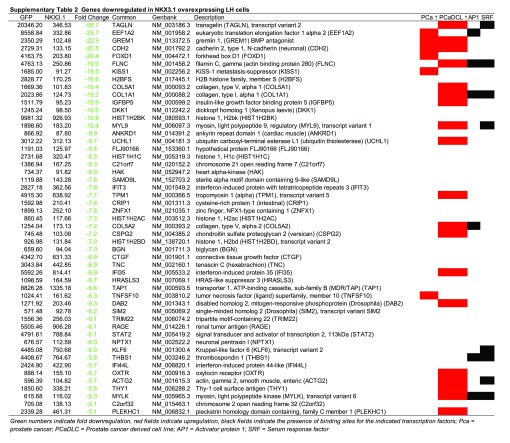


**Figure:**
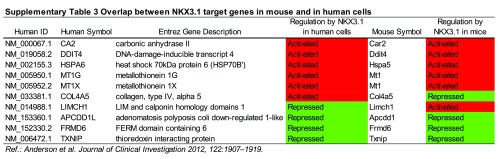


**Figure:**
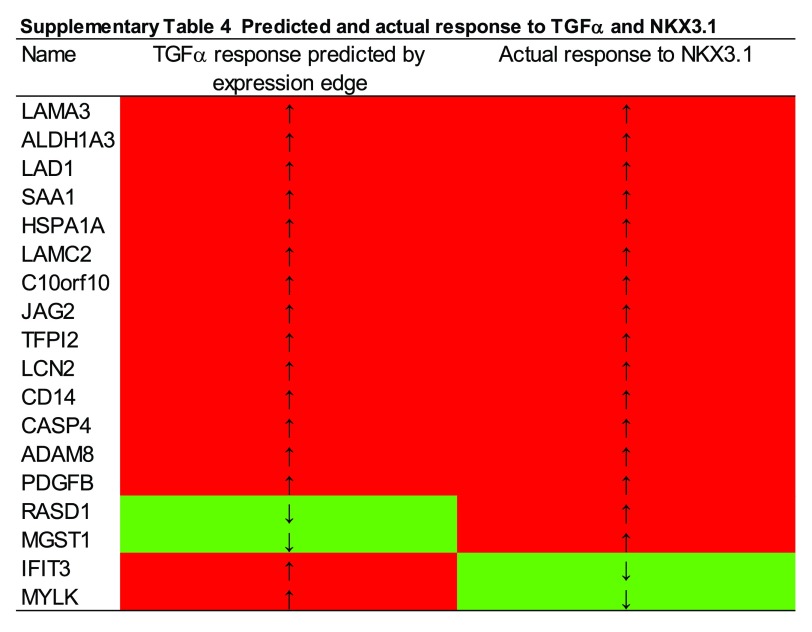


**Figure:**
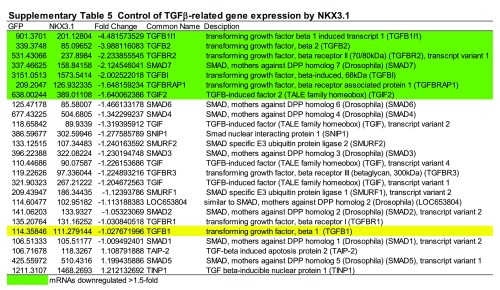


**Figure:**