# Supplementary material for: Systems analysis of the prostate tumor suppressor NKX3.1 supports roles in DNA repair and luminal cell differentiation
Source: F1000Res. 2014 Dec 18;3:115. Originally published 2014 May 21. [Version 2] doi: 10.12688/f1000research.3818.2 (PMC4141641; doi:10.12688/f1000research.3818.2)
Supplement: Peer review history

# Peer review: Referee response for version 1

**DOI:** 10.5256/f1000research.4090.r5066

**Published:** 2014-06-26

**Authors:** Philip D. Anderson

**Affiliations:** 1 Department of Biological Sciences, Salisbury University, Salisbury, MD, USA

This manuscript presents results from multiple experiments identifying and characterizing the interactome of NKX3.1 in immortalized human prostate epithelium (LH) and human prostate cancer cells (LNCaP). The manuscript is well-researched and well-written. This research helps to fulfil an unmet need by the research community, which is to explain the role of NKX3.1 in the prostate epithelium. In that sense, the results are timely. I have only a few comments, questions or points to the authors that need to be addressed.

1. Explain why there are no error bars in Figure 4A?
2. The authors mentioned that they used the 'affy' package in Bioconductor to preprocess their microarray data. Please indicate the affy package version, Bioconductor version, and R version in the methods.
3. The affy package should be cited in the bibliography with this citation: Gautier, L., Cope, L., Bolstad, B. M., and Irizarry, R. A. 2004. affy---analysis of Affymetrix GeneChip data at the probe level. Bioinformatics 20, 3 (Feb. 2004), 307-315.
4. The authors used a heteroscedastic t-test to infer differences in gene expression in their microarray studies. In the methods, please indicate the multiple testing correction that was applied.
5. In *NKX3.1-induced transcriptional program, *final paragraph: There are 331 mRNAs changed >= 3-fold. But in *Pathway analysis*, first paragraph, the 3x dataset is 357 genes. Please explain how these datasets are different?
6. Please indicate the kDa ladder on Figure 3B.

I have read this submission. I believe that I have an appropriate level of expertise to confirm that it is of an acceptable scientific standard.

# Response

**Published:** 2014-12-11

**Authors:** Dieter A Wolf

**Affiliations:** Tumor Initiation & Maintenance Program / Degenerative Disease Program, Sanford Burnham Prebys Medical Discovery Institute, USA

1. *Explain why there are no error bars in Figure 4A?*
  This figure shows p values from the pathway analysis, which do not have variations.
2. *The authors mentioned that they used the 'affy' package in Bioconductor to preprocess their microarray data. Please indicate the affy package version, Bioconductor version, and R version in the methods.*
  R-version was 2.10.1 and the bioconductor version 2.5 with the appropriate affy package downloaded by the package manager.
3. * The affy package should be cited in the bibliography with this citation: Gautier, L., Cope, L., Bolstad, B. M., and Irizarry, R. A. 2004. affy---analysis of Affymetrix GeneChip data at the probe level. Bioinformatics 20, 3 (Feb. 2004), 307-315.*
  The reference has been included.
4. *The authors used a heteroscedastic t-test to infer differences in gene expression in their microarray studies. In the methods, please indicate the multiple testing correction that was applied.*
  Due to the low number of replicates (2 per time point), no correction was applied. Our rationale is that we initially cast a wider net by basing our gene lists on uncorrected p values. The lists are subsequently validated by the orthogonal method of pathway analysis, where we make the assumption that random changes would not manifest as enriched pathways.
5. *In NKX3.1-induced transcriptional program, final paragraph: There are 331 mRNAs changed >= 3-fold. But in Pathway analysis, first paragraph, the 3x dataset is 357 genes. Please explain how these datasets are different?*
  We apologize for this error, which was corrected. The 3x dataset of 357 genes was used for the analysis.
6. *Please indicate the kDa ladder on Figure 3B.*
  Unfortunately, the original film of this blot which was run in 2007 could not be ascertained. However, Fig 6C shows the expression of GFP and NKX3.1 from the same virus with size markers. The relative sizes of the GFP and NKX3.1 bands are consistent between Fig. 3B and Fig. 6C.

# Peer review: Referee response for version 1

**DOI:** 10.5256/f1000research.4090.r4848

**Published:** 2014-06-20

**Authors:** Kemal S. Korkmaz

**Affiliations:** 1 Department of Bioengineering, Ege University, İzmir, Turkey

Authors have extensively studied the tumor suppressor function of NKX3.1 using multiple gene expression profiling approaches with validations. Eventually, they have demonstrated the NKX3.1 interactome, which revealed a complex pattern of interactions with DNA damage repair proteins including Ku70, PARP1 and XRCC5 in addition to other transcriptional regulators such as ILF2 and BANF1.

To perform their research, they have used recognized approaches for the analysis of the gene expression patterns upon ectopic expression of NKX3.1 in immortalized human prostate epithelial cells with a basal phenotype, which revealed a rapid and extensive re-programming with 158 mRNAs changing higher than 5-fold and 331 mRNAs changing higher than 3-fold. Since the data obtained and presented here is consistent with the previous reports, especially Bowen *et al.* as well as Erbaykent-Tepedelen *et al.*, suggest that the NKX3.1-induced gene signature is similar to the gene expression pattern found in early human prostate cancers. Therefore, the data give insights about the requirement of the NKX3.1 as a key driver of luminal cell differentiation, its loss allows luminal cells to dedifferentiate into a state with higher proliferative capacity leading to the increased genetic heterogenity, perhaps augmented by concurrent defects in DNA damage repair pathways.

I have read this submission. I believe that I have an appropriate level of expertise to confirm that it is of an acceptable scientific standard.

# Response

**Published:** 2014-12-11

**Authors:** Dieter A Wolf

**Affiliations:** Tumor Initiation & Maintenance Program / Degenerative Disease Program, Sanford Burnham Prebys Medical Discovery Institute, USA

Since no specific concerns were raised, we thank the reviewer for his efforts!
